# Population Dynamics of the Widespread Alien Decapod Species, Brown Shrimp (*Penaeus aztecus*), in the Mediterranean Sea

**DOI:** 10.3390/ani15040561

**Published:** 2025-02-14

**Authors:** Mehmet Cengiz Deval, Tomris Deniz

**Affiliations:** 1 Faculty of Fisheries, Akdeniz University, 07058 Antalya, Turkey; 2 Faculty of Water Science, İstanbul University, 34134 İstanbul, Turkey; tomris@istanbul.edu.tr

**Keywords:** growth, age, eastern Mediterranean, spawning, *Epipenaeon ingens ingens*, sea turtles, gear selectivity, turtle excluder devices

## Abstract

This study investigated the population dynamics, growth, reproduction, and parasitism of *Penaeus aztecus* over a 27-month period in Antalya Bay (Eastern Mediterranean). *P. aztecus* was the most abundant shrimp species, comprising 53.4% of the collected shrimp specimens. Abundance varied seasonally, with peak densities in summer. Males had a smaller mean carapace length (CL) of 25.8 mm compared to females at 30.2 mm. Females dominated larger size classes, with all individuals ≥ 46 mm CL being female. The sex ratio was balanced at 1:1, with no seasonal variations. Spawning occurred year-round, peaking in June, late summer, and November. Females displayed four ovarian stages, with the first mature size (FMS) at 36 mm CL. Parasitism by *Epipenaeon ingens* reduced the density of mature females, with 42% of potential spawners failing to develop gonads. The prevalence of parasitism showed seasonal variation and was inversely correlated with sea surface temperature (SST). Marine recruitment occurred from June to November, with a peak between July and September. Growth analysis revealed faster rates in females, while males reached a smaller maximum size. This study also identified inefficiencies in gear selectivity, with many juvenile shrimp (below recruitment size) being retained. Trawl efficiency improved with the use of turtle excluder devices (TEDs), which reduced bycatch of nontarget species, such as loggerhead turtles and cartilaginous fishes. The instantaneous total mortality rate (Z) ranged from 0.658 to 0.026 month⁻^1^ for male shrimp and from 1.00 to 0.014 month⁻^1^ for female shrimp, with survival sharply declining after recruitment, leaving only about 3.6% of individuals surviving beyond 10 months.

## Simple Summary

This study focuses on the brown shrimp, an Atlantic species that has spread rapidly into the Mediterranean since its first discovery in 2009 and has become an economically important crustacean. Despite increased distribution, landings, and aquaculture efforts, comprehensive information on its population dynamics, such as reproduction, recruitment, age, growth, selectivity, and mortality, is still lacking. Length–frequency distributions over a 27-month period from surveys and commercial bottom trawls reveal sexual dimorphism, with faster growth observed in females. These results improve understanding of reproduction, spatiotemporal migration, growth, mortality dynamics, and trawl selectivity of brown shrimp, providing valuable insights for sustainable fisheries management in the Mediterranean.

## 1. Introduction

The brown shrimp *Penaeus aztecus* (Ives, 1891), an economically significant species, is widely distributed from Massachusetts to around the tip of Florida and throughout the Gulf of Mexico to the northwestern Yucatan Peninsula [1]. Its highest density occurs along the coasts of Louisiana as well as Texas and Mississippi [2]. In the Gulf of Mexico, approximately 1500 U.S.-flagged shrimp trawlers operate under federal permits [3], while Mexico conducts intensive fishing in the region with around 316 industrial trawlers (18–25 m) and over 2500 artisanal vessels (6–9 m) using small trawls and other gear, primarily targeting *P. aztecus* [4]. Annual landings of wild-caught brown shrimp in 2015 amounted to 48,761 t with a commercial value of $165.4 million in the U.S. [5]. Fishery is also one of the most important sectors in Mexico, accounting for up to 90% of the country’s total shrimp landings [6].

In the Atlantic populations in the Gulf of Mexico, adults of *P. aztecus* spawn in offshore locations [6], post-larvae migrate into estuaries [7,8], small juveniles settle and grow in inshore bays, and larger juveniles subsequently move back offshore to mature into adults [9].

In the Mediterranean, *P. aztecus* was first recorded in Antalya Bay (eastern Mediterranean) in 2009, probably introduced via ballast water [10], and its distribution along the Egypt–France line in the northeastern Mediterranean has rapidly expanded over the last 15 years [11]. *P. aztecus* is now one of the most widespread alien decapod species in the Mediterranean Sea [12], with distribution areas including Egypt, Israel, Turkey, Greece, Montenegro, Italy, Malta, and France [11]. Since 2016, shrimp farmers have been cultivating the species using post-larvae and juveniles collected from the wild in the Nile estuary, Egypt [13]. Different colonization scenarios have been proposed for the rapid spread of *P. aztecus* in the Mediterranean. The first record of the species in the Mediterranean Sea attributed its presence to the likely introduction of larvae through ballast water discharge [10]. Subsequently, some researchers [12,14,15] proposed that the species may have also spread due to escapes from unreported or illegal shrimp farming operations. However, Froglia and Scanu [16] argue that, aside from deliberate introductions of *P. aztecus*, for which official documentation may exist, the introduction pathways in other cases remain speculative and based on informed assumptions. In the Gulf of Mexico, a study conducted between 1978 and 1980 [17] involved the release of over 71,000 tagged brown shrimp at various offshore fishing sites. The study reported a recapture rate of over 12%, with two specimens notably recaptured after 430 and 400 days at sea, having traveled distances of 596 km and 528 km, respectively, from their release points. Considering this natural dispersal capacity, it may represent the primary mechanism behind the rapid spread of *P. aztecus* in the Mediterranean. However, the numerous records of *P. aztecus* from distant locations, such as the North Tyrrhenian Sea and the Gulf of Lion, suggest that multiple introduction events have contributed to its spread in the Mediterranean Sea [16].

On the eastern Mediterranean coast of Turkey (Antalya, Mersin, and İskenderun Bays), where *P. aztecus* was first recorded in the Mediterranean, it has become the most important commercial shrimp species, with its price ranging between EUR 20 and 30 per kilogram, depending on the season. The species is also one of the most significant new penaeid species for the commercial shrimp fishery off the eastern coast of Egypt [13]. Along the southern Italian coasts (Sicily, Calabria, Catania, and Scoglitti), it is commonly trawled and marketed at prices ranging from EUR 15 to 20 per kilogram [18,19]. Despite the expansion of the range and the rapid increase in the density of *P. aztecus* in the Mediterranean Sea, which has created a new resource for small-scale fisheries and aquaculture, no studies have yet been conducted on the population dynamics of the species, aside from the initial records and identifications. However, the parasitic isopod *Epipenaeon ingens ingens* (Nobili, 1906) poses a significant threat to the wild stock of *P. atecus*, affecting both the reproductive potential of the shrimp and their commercial value [11].

The objective of this study is twofold. First, it aims to provide baseline data for the sustainable management of the Mediterranean population of *P. aztecus*, covering aspects such as abundance, spawning, marine recruitment, growth, selectivity, and mortality. Second, it seeks to facilitate a comparison with the Atlantic population.

## 2. Materials and Methods

### 2.1. Study Area and Sampling

**Figure 1:**
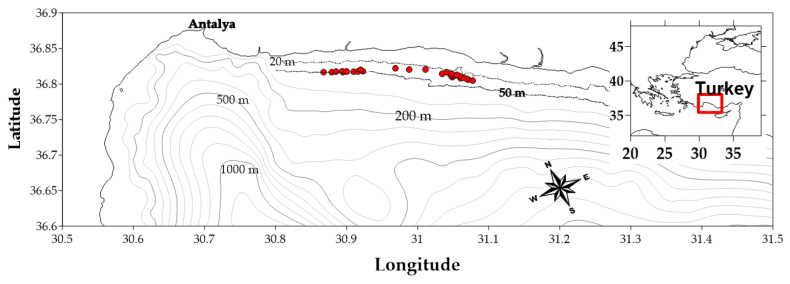
Trawl sampling tracks in the Antalya Bay (eastern Mediterranean). The red dots indicate the starting point of each trawl haul.

A monthly sampling program was carried out during the nighttime onboard “R/V Akdeniz” (overall length 26.5 m, 160 GRT, engine power 670 kW) using a standard otter trawl from March 2019 to November 2021 in Antalya Bay (eastern Mediterranean Sea) (Figure 1). Due to the negative impacts of the global COVID-19 pandemic, sampling was not possible from March to May and from August to October 2020. To facilitate comparison, the initial 12-month period was designated as the pre-COVID phase (March 2019 to February 2020; Survey 1), while the subsequent 13-month period was classified as the post-COVID phase (November 2020 to November 2021; Survey 2) (Table 1). However, samples were obtained from commercial fisheries in March and April 2021 and used only to monitor gonadal maturation. The study area is situated at a distance of between two and three nautical miles from the coastline, at a depth of between 25 and 50 m. Towing duration ranged from 50 to 200 min, and the average towing speed was 2.6 knots.

As the beach in the sampling area is also one of the most important nesting sites for loggerhead sea turtles (*Caretta caretta*), a grid-based dual selection system in the codend was used to avoid sea turtles and large cartilaginous species. A grid “Super Shooter turtle excluder device (TED)” was mounted in front of the codend of the trawl net (the distance between the TED and the beginning of the codend was 5 m) [20,21], and the dimensions of the TED were 120 cm × 90 cm, with 9 cm spacing between the bars. The angle of the TED was approximately 45°, with the exit hole pointing in a downward direction. The experiment utilized a grid cover (GC) made of polyamide with 22 mm diamond mesh and measuring 5 m in length, positioned over the outlet in front of the grid to collect fish (with large cartilages), sea turtles, and unwanted objects escaping from that area. A codend cover (CC: polyamide with 22 mm diamond mesh and measuring 5 m in length) was placed over the codend (CE: polyethylene with 44 mm diamond mesh) to collect codend escapees. To prevent a masking effect, the codend cover was supported by two PVC hoops, and TED was secured with detachable floats [22]. Further details and figures on the codend and TED can be found in [20,21].

After each haul at night, the total catch from each of the three compartments (codend, codend cover, and grid cover) was sorted and weighed separately, and the length of the species of interest was measured to the nearest 0.5 cm for fish and the nearest 0.1 mm for shrimp. All shrimp species were promptly frozen in seawater and subsequently transported to the laboratory for further biological examination.

### 2.2. Data Analysis

The percentage of moon illumination for each sampling night and the monthly average sea surface temperature (SST, °C) were obtained from a website [23] and the Meteorological Regional Directorate in Antalya, respectively [24]. In the laboratory, the sex of 5790 specimens (N_TOT_) of *P. aztecus* with a total mass of 96.53 kg was determined from the secondary sexual characteristics (presence of telycum in female “F” and petasma in male “M”) by macroscopic observations. Carapace length (CL, 0.1 mm) was measured, and the length–frequency standardized distributions (LFSD, number of specimens standardized in one km^2^) were represented by sex. The sex ratio (SR = N_F_/N_TOT_) was calculated for the whole population, and the Chi-square (χ^2^) test was used to assess the predominance of the females. The similarity in the length frequency between sexes was tested using the Kolmogorov–Smirnov (K-S) test at *p* = 0.05.

In order to evaluate the marine recruitment of *P. aztecus*, individuals smaller than 28 mm CL for both sexes in each month were considered to be young-of-the-year specimens (N_REC_) [11]. A total of 3861 *P. aztecus* individuals were examined from March 2019 to May 2021 to determine the presence of *E. i. ingens* in their branchial chambers due to the fact that the parasitic isopod induces gonadal sterilization in female shrimps [8]. Although females with a CL ≥ 34 mm were considered potential spawners (F_PS_), only uninfected mature females (F_MAT_) were examined monthly (when possible 40 specimens per month) to assess the ovarian development. After measuring CL and total weight (TW, 0.01 g), mature females were dissected, ovaries extracted, and weighed (GW, 0.0001 g). The gonadosomatic index (GSI) was calculated for each month using the following formula: GSI = GW/TW × 100. A new four-stage key was developed using the five-stage classification of [25] and the three-stage classification of [26]. This classification was used to assess the developmental stages macroscopically according to the external characteristics of the gonads, including ripening position, degree of turgidity, size, and color, as follows: undeveloped stage (St.I), developing stage (St.II), early to almost mature stage (St.III), and mature stage (St.IV). In addition, the occurrence of each maturity stage was determined for 2 mm length classes of uninfected females between 20 and 50 mm CL during the period of maximum gonadal activity (between June and August). To calculate size at first maturity (CL_%50_: the CL at which 50% of the females were mature), specimens with ovaries (St.III and St.IV) were considered mature, while the first two stages were considered immature. The probability of maturity was modeled using a logistic curve, r(CL) = [exp (v_1_ + v_2_.CL)/1 + exp (v_1_ + v_2_.CL], using the software CC2000 [27], where r(CL) is the proportion or probability of reproductive individuals for each size class (CL), v_1_ and v_2_ are regression parameters to be estimated.

The swept area (km^2^) was calculated according to the wing spread of the trawl (17.5 m) and the start-end points algorithm in the AdriaMed Trawl Information System (ATrIS) [28]. The biomass (BI, kg. km^−2^), five abundance indices (overall density (D_TOT_), density of females (D_F_), density of potential spawner females (D_FPS_), density of mature females (D_MAT_), and density of recruits (D_REC_)), and two fractions (D_REC_/D_TOT_ and D_MAT_/D_TOT_) per haul were estimated from the pooled number (N) and weight (kg) of individuals of three compartments in the swept area using the ATrIS v.2.1 software (version 2.1) (Table 1). The null hypothesis of no difference and/or significant correlation in all these indices and fractions among seasons and surveys was tested using two-way MANOVA and non-parametric (Spearman’s “rho”) correlation, respectively. Furthermore, significant trends in all these values with regard to the SST and moonlight % were investigated through the use of Spearman’s correlation analysis for both surveys.

The CL/TW relationships were estimated by fitting an exponential curve (TW = a × CL^b^) converted to its logarithmic expression (log W = log a + b log CL), where a is the intercept and b is the slope. A Student *t*-test was used to determine the significance of differences between the isometric growth (b = 3) and the estimated b value of the equation. The monthly LFSD of *P. aztecus* for both sexes was utilized to estimate the modal mean CL of all cohorts over a period of 27 months (Supplementary Table S1), employing the Bhattacharya method within the FISAT II v.1.2.0.2 software package [29]. The relative age, tr + Δt (time period (months/years) after marine recruitment, tr), of *P. aztecus* in the study was estimated, as absolute ages could not be determined. This limitation arose because the migration to the estuary, residence there, and subsequent return to the sea encompasses a significant part of the life span of *P. aztecus*, which was not covered in this study (see detail in the Section 4). The Bhattacharya procedure was performed a second time using the pooled LFSD data sets for 12 months from each sex (Supplementary Tables S2 and S3) to estimate the relative age of shrimps, and the modal lengths for each cohort were then linked through subjective interpretation of a graph. The outputs of the “linking of means” were stored in two distinct formats: (i) growth increment data and (ii) length-at-(relative)-age data for analysis with the “length-at-age routine”. The seasonal growth oscillations were found to be significant and strong for female (C < 0.70) and male (C < 0.60). According to the Gulland and Holt plot, two new parameters (C and ts) were added and fitted to the “length-at-age” routine for re-estimation. Ultimately, a seasonalized von Bertalanffy growth function (VBGF) was obtained for both sexes [30].

$$L_{t}=L_{inf}(1-\exp\left(-K\left(t-t_{0}\right)+St_{s}+St_{0}\right)$$

$$St_{s}=\left(\frac{CK}{2\pi }\right)x\;Sin\left(t-t_{s}\right)\;\mathrm{and}\;St_{0}=\left(\frac{CK}{2\pi }\right)\times Sin\left(t_{0}-t_{s}\right)$$

where t = relative age (tr + Δt), L_t_ = length at age t (mm), L_inf_ = asymptotic CL (mm), K = growth coefficient (per year), C = amplitude of oscillations, t_0_ = age of the shrimp at zero length (year), and t_s_ = starting time of the sinusoid growth oscillation. The t_s_ was replaced with WP (winter point, which is the period when growth is slowest) as WP = t_s_ + 0.5.

Three different selectivity processes were performed during the analysis. (*i*) the grid selection (u(TestCover2) Cover1) was estimated by comparing the retained catch in the CE and the escaped catch in the CC with the excluded catch with grid in the GC; (*ii*) the codend selection (uTestCover2) was estimated by comparing the retained catch in the CE with the escaped catch in the CC; and (*iii*) the combined system selection (uTestCover1Cover2) was estimated by comparing the retained catch in the CE with the excluded catch with the grid in the GC plus the escaped catch in the CC. The software tool SELNET v.10 [31] was employed for the analysis, with 1000 bootstrap iterations utilized to estimate the confidence intervals (CIs) (for more detailed information on system covers and TED design, see [20,21,31,32,33]).

Total mortality (Z) was calculated for each sex using the pooled LFSD data (Supplementary Tables S2 and S3), and the “survival rate” (S) was determined according to the method outlined by [34]:

$$S=N(t+\Delta t)/N\left(t\right)=exp\left(-Z\right)$$

where t represents July, the month of highest population density; N(t) denotes the population size in July; and Δt refers to the time period after July. As a consequence of mortality, the number of survivors in the cohort declines continuously. If the parameter Z is assumed constant over the cohort’s lifetime, the number of survivors at any given time (t + Δt) can be expressed using the “exponential decay model”. This is represented by the following mathematical equation, which was previously used by [35]:

N(t + Δt) = N(t) exp[−Z(t + Δt)],

where N(t + Δt) is the number of survivors from a cohort attaining the relative age t + Δt.

## 3. Results

### 3.1. Shrimp Species Composition

A total area of 5.62 km^2^ was swept over a 27-month period, and seven shrimp species belonging to the family Penaeidae were collected. *P. aztecus* was the most abundant species (53.4%), with 2782 female (N_F_) and 3008 male (N_M_) individuals. The next most prevalent species, in descending order, were *Penaeus pulchricaudatus* (Spence Bate, 1888) at 25.6%, *P. semisulcatus* (De Haan, 1844) at 11.1%, and *Metapenaeus monoceros* (Fabricus, 1798) at 9.2%. *P. kerathurus* (Forskȧl, 1775), *Metapenaeopsis aegyptia* (Galil & Golani, 1990), and *P. hathor* (Burkenroad, 1959) collectively accounted for only 0.5% of the shrimp abundance. The monthly average SST values exhibited a range between 17.1 °C (February 2020) and 29.5 °C (August 2021) with a mean of 22.96 ± 4.6 °C (Table 1); no statistically significant difference was observed between the two surveys (t_(26)_ = 94.942, *p* = 0.996).

### 3.2. Population Structure

Some of the obtained and computed basic parameters are presented in Table 1, while the monthly LFSD (ind. km^−2^) during the sampling period, separated by sex, is shown in Supplementary Table S1. The CL of 3008 male shrimps ranged from 14 mm to 44 mm, with a mean of 25.8 ± 4.1 mm. Larger sizes were uncommon, with only 12 individuals (0.07%) having a CL of 40 mm or greater, and 93 individuals (3.1%) measuring at least 36 mm. Among 2 782 female shrimps, the CL ranged from 14 mm to 64 mm, with a mean of 30.2 ± 6 mm. Similarly, larger females were rare, as only 6 individuals (0.2%) had a CL of 48 mm or greater, and 71 individuals (2.5%) measured at least 42 mm. The difference of 4.4 mm CL (95% CI [5.07, 4.19]) was statistically significant (t_(5785)_ = 20.80, *p* = 0.001). The K-S test also showed a significant difference between the sexes for the LFSD (D_OBS_ = 0.424 > D_EST_ = 0.023; *p* = 0.001). Females dominated the population from 30 mm CL onward (61.5%), with the percentage increasing to 89% at 40 mm CL. All individuals greater than 46 mm CL were females. The total abundance (D_TOT_) exhibited considerable variability, ranging from a low of 32 ind. km^−2^ in May 2021 to a high of 5559 ind. km^−2^ in July 2019. Seasonal variation in D_TOT_ was highest in summer (2137 ind. km^−2^) and lowest in spring (156 ind. km^−2^), with a gradual decrease observed in winter (299 ind. km^−2^). The D_TOT_ and BI indices demonstrated statistically significant discrepancies between seasons (*p* = 0.000 and *p* = 0.011) and months (*p* = 0.000 and *p* = 0.001), while no statistically significant differences were observed between the surveys (*p* = 0.419 and *p* = 0.171) (Table 2). Spearman’s rho correlation revealed a strong, statistically significant correlation between SST and both D_TOT_ (r = 0.724, *p* = 0.000) and BI indices (r = 0.630, *p* = 0.000). In contrast, moon illumination showed no statistically significant relationship with these two indices (Table 3).

### 3.3. Prevalence of E. ingens ingens

**Figure 2:**
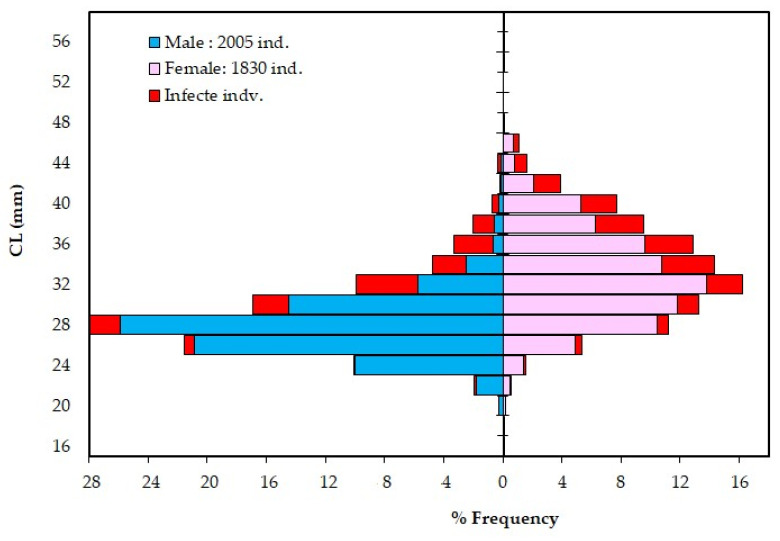
Length frequency distributions of infected and non-infected shrimp by carapace length (CL) and by sex of *Penaeus aztecus* sampled in Antalya Bay (a 64 mm CL female not included).

A total of 3835 *P. aztecus* were inspected between March 2019 and April 2021. Of these, 338 males (16.9%) and 387 females (21.1%) were found to be infected with the bopyrid parasite *E. ingens ingens*. The infection rates of male and female shrimp did not differ significantly (χ^2^ = 0.019, df = 1, *p* > 0.05). No parasites were identified in shrimp specimens less than 20 mm CL. Therefore, the prevalence was exceedingly low at the extensive recruitment in July (1.23%), but it increased with the growth of the recruiting class in consecutive months, reaching 58.97% in March in the overall population. The infestation rate was found to be 5.4% in young-of-the-year specimens (CL < 28 mm, 1585 individuals), while it increased to 39.6% in older specimens (CL ≥ 34 mm, 814 individuals) (Figure 2). The analysis (except for classes with a single individual) revealed a highly positive correlation between infestation and host size for both sexes (M: r = 0.899, *p* = 0.01; F: r = 0.844, *p* = 0.01). The prevalence exhibited notable statistical fluctuations in accordance with the seasons (*p* = 0.002), whereas no substantial discrepancies were observed between surveys (*p* = 0.983). A strong negative correlation was observed between SST and prevalence in the overall population (r = −0.794, *p* = 0.000).

### 3.4. Reproduction Pattern

**Figure 3:**
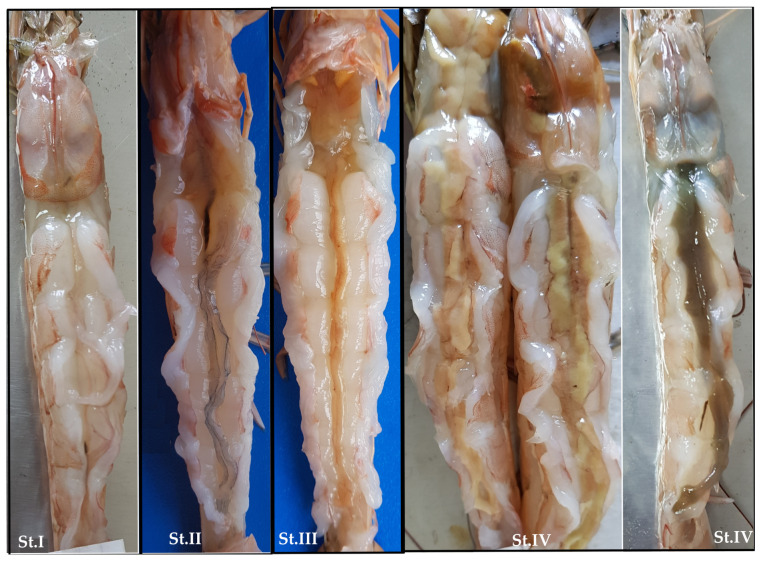
The four macroscopic developmental stages of the ovary in female *Penaeus aztecus*.

**Figure 4:**
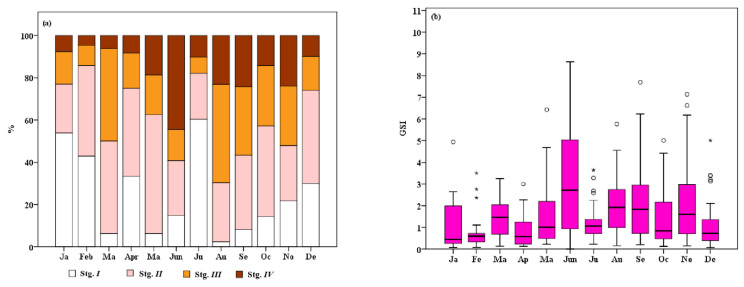

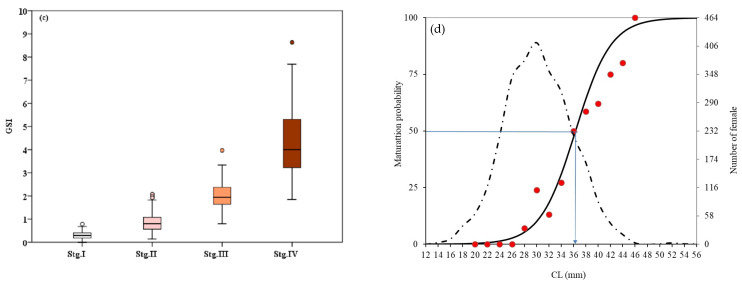
(**a**) Pooled monthly percentage distribution of the four ovarian developmental stages of *Penaeus aztecus* from Antalya Bay (eastern Mediterranean) during the study period, from March 2019 to November 2021. (**b**) Monthly pooled oscillation of the gonadosomatic index (GSI) for standard sizes (CL ≥ 34 mm), with extreme values indicated by “o” and high extreme values by “*”. (**c**) Overlap of GSI values across the four ovarian stages. (**d**) First maturation size (CL_50%_) for female specimens.

Despite a minimum of 0.43 (in April) and a maximum of 0.63 (in December) fractions during the study, the sex ratio (SR) did not differ significantly from the 1:1 ratio (χ^2^, *p* > 0.05). The data revealed no statistically significant influence of season (*p* = 0.467), month (*p* = 0.475), or survey (*p* = 0.788) on the SR (Table 2). In addition, 42% of the potential spawner females, representing 32% of the total female shrimp with an average of 158.8 ± 228 ind. km^−2^, did not develop gonads over the course of the year as a consequence of the influence of *E. ingens*. Therefore, the density of mature females without parasites (D_MAT_ = 92.9 ± 206 ind. km^−2^) was only 18.6% of all females. Season (*p* = 0.659), month (*p* = 0.527), and survey (*p* = 0.059) had no statistically significant effect on the D_MAT_ index (Table 2). The remaining 285 parasite-free mature females (between 34 and 64 mm CL) were examined using macroscopic criteria to identify four distinct ovarian developmental stages (Figure 3 and Figure 4a):

Stage I (Undeveloped): The ovaries are extremely thin, translucent, and difficult to remove. They are not visible externally.

Stage II (Developing): The posterior lobe diameter exceeds that of the dorsal abdominal artery. The ovaries change color, ranging from pinkish to light yellow or greenish hues.

Stage III (Early to Late Mature): The posterior lobe enlarges, increasing in girth. At the advanced stage, the ovary develops further, with the anterior, middle, and posterior lobes filling the cephalothorax. The ovary typically appears yellowish to orange, with occasional brownish chromatophores visible on the surface.

Stage IV (Mature): The ovaries are clearly visible through the exoskeleton, exhibiting well-developed lobes and a fully distended posterior lobe. The color varies from bright orange to dark orange or even very dark orange.

The observation reveals that four distinct ovarian stages are present throughout all months. The consistent presence of maturing (Stage III) and mature (Stage IV) ovaries across months indicates that *P. aztecus* engages in continuous breeding year-round. Figure 4b illustrates the oscillatory pattern of the gonadosomatic index (GSI) values on a monthly basis over a one-year period, with peaks in spawning activity observed in June, August to September, and November. The results of the one-way ANOVA test indicated that both months (F_(11, 292)_ = 4.279, *p* = 0.000) and seasons (F_(3, 292)_ = 6.034, *p* = 0.001) had a significant impact on the GSI of *P. aztecus*. Significant differences in CL, TW, GW, and GSI were observed across the maturation stages (Table 4). The CL and TW were significantly lower in Stage I (*p* = 0.05) compared to the other stages. However, GW and GSI values exhibited statistically significant differences across all four stages. The GSI was strongly correlated with the maturation stage, with mean values ranging from 0.32 in Stage I to 4.4 in Stage IV (Figure 4d). Overlapping GSI values were observed between successive stages, but all females with a GSI above 3.94 were classified as being in Stage IV.

Female specimens of *P. aztecus* with ovaries in Stage III and IV were categorized as mature shrimps, while those in Stage I and II were classified as immature. This classification was used to calculate the first maturity size (FMS), defined as the CL at which 50% of the individuals are mature. The FMS for females was estimated to be ≌36 mm CL (Figure 4d). The smallest females observed in Stage IV and III were 30 mm CL and 28 mm CL, respectively.

### 3.5. Marine Recruitment

**Figure 5:**
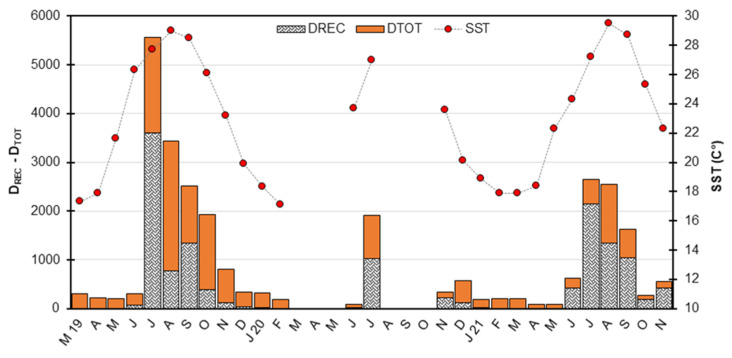
Monthly fluctuations in the density of recruits-of-the-year (D_REC_), total density (D_TOT_), and sea surface temperature (SST) observed during the surveys.

**Figure 6:**
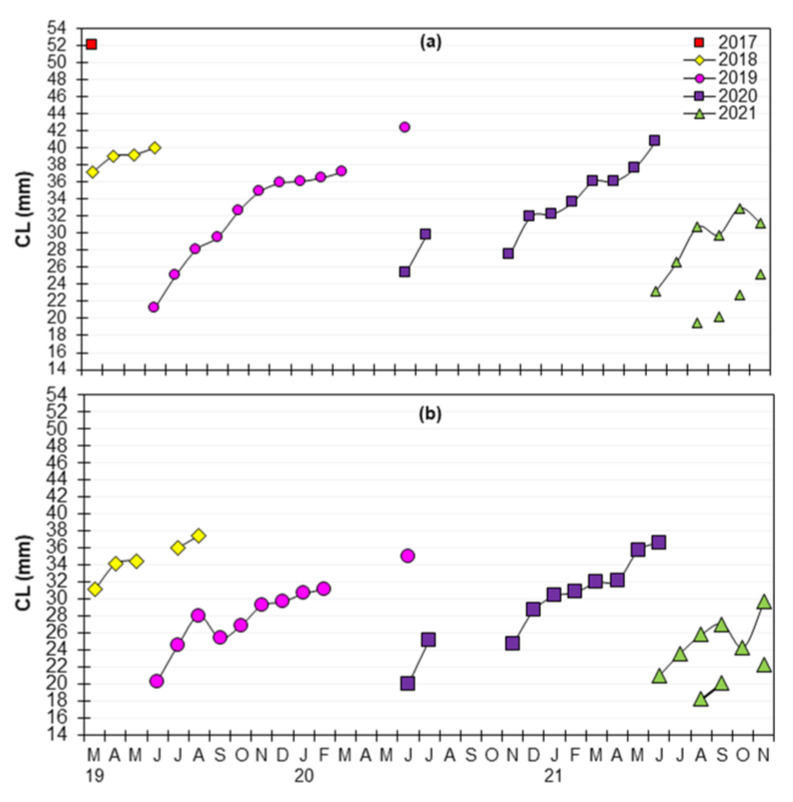
The mean CLs of the identified modal groups of five cohorts of *Penaeus aztecus* on a modal progression analysis for female (**a**) and male (**b**) over a 27-month period in the Antalya Bay.

Marine recruitment of *P. aztecus* always began in June with small groups and lasted until November, peaking between July and September (Figure 5). In the first and second surveys, approximately 86% and 80% of the annual recruitment, respectively, occurred during this peak period, with maximum values observed in July (50% and 35%). Due to the pandemic-related closure, samples were collected only in June and July between March and October 2020, and these two months had recruitment timings before and after the pandemic (Table 1). In autumn 2021, an influx of recruit groups resulted in a bimodal recruitment pattern (Figure 6). However, no statistically significant difference in the D_REC_ index was found between surveys (*p* = 0.927). Conversely, season (*p* = 0.000) and month (*p* = 0.042) significantly influenced the D_REC_ index (Table 2). A strong positive correlation was observed between SST and the D_REC_ index (r = 0.763, *p* = 0.000), indicating that marine recruitment was associated with warmer months. In contrast, significant negative correlations were found between SST and mean CL (r = −0.677, *p* = 0.000) and TW (r = −0.741, *p* = 0.000), reflecting the recruitment of smaller individuals into the area (Table 3).

### 3.6. Length–Weight Relationships, Growth, and Age

**Figure 7:**
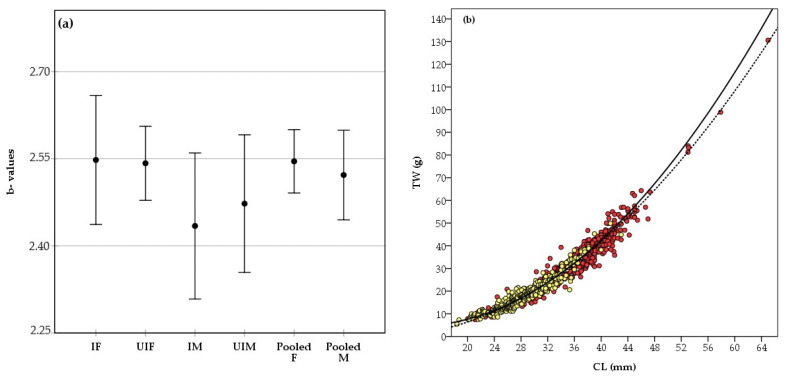
(**a**) Carapace length (CL) to total weight (TW) regression slopes (b ± confidence intervals) for *Penaeus aztecus* by group: infected females (IF) and - male (IM), uninfected females (UIF) and - male (UIM), pooled female (F) and - male (M). (**b**) CL/TW relationships for both sex (straight line and yellow circles for male, dashed line and red circles for female).

Figure 7a compares the slopes (b values) of the regression lines for the TW/CL relationships in both sexes, considering the presence or absence of infection. Comparisons revealed no statistically significant differences between the slopes at the 95% CI, as the CIs overlapped. Analyses using pooled data (infected and uninfected specimens) for both sexes were performed, indicating a negative allometric growth pattern (Figure 7b).

Female→: TW = 0.0035 × CL^2.546^→r^2^ = 0.933→N:611 (20 − 65 mm CL)

Male→: TW = 0.0038 × CL^2.522^→r^2^ = 0.914→N:388 (18 − 43 mm CL)

**Figure 8:**
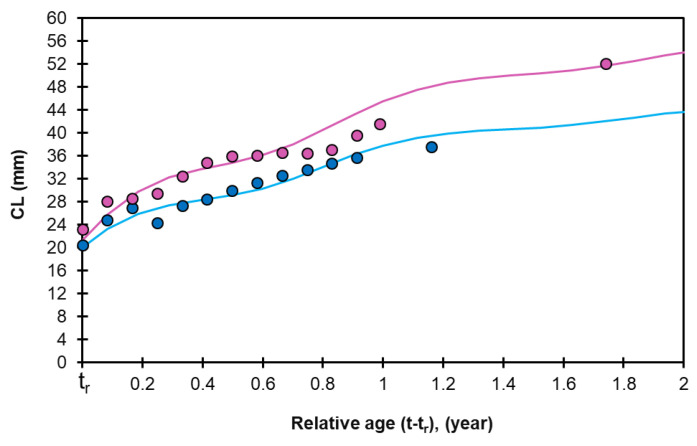
Seasonally oscillating von Bertalanffy growth curves fitted to the observed mean carapace length (CL) at relative age for both sexes of *Penaeus aztecus*. The pink line represents the estimated growth curve for females, with pink circles indicating the observed mean CL values (mm). The blue line represents the estimated growth curve for males, with blue circles marking the observed mean CL values (mm).

Modal progression analysis applied to the monthly standardized length–frequency distribution of *P. aztecus* identified five age cohorts (2017–2021), with a maximum of two-year modes for both sexes (Figure 6). Pooled data revealed that the mean CL of female recruits in June was 23.2 mm, increasing to 34.1 mm by December and 41.5 mm the following June. Only 2.8% of females reached their second year after recruitment, attaining a CL of ≥42 mm. By 21 months post-recruitment, the oldest modal group (1.74 years of relative age) from the 2017 cohort was observed with a mean CL of 52 mm in March 2019 (Figure 7a). Identifying modal groups was more challenging for male shrimps, which exhibited slower growth compared to females. Male recruits had a mean carapace length (CL) of 20.4 mm in June, increasing to 29.2 mm by December and reaching 35.7 mm the following June. The oldest male group, with a relative age of 1.16 years and a mean CL of 37.5 mm, was observed 14 months after recruitment. Notably, only 3.1% of males survived to their second year, attaining a CL of ≥36 mm (Figure 6 and Figure 8).

The identified age groups (modal groups) and their abundance showed that increments between consecutive groups generally followed a decreasing trend. The estimated growth parameters for females (CL_inf_ = 58.5 mm, K = 1.05 year⁻^1^, and C = 0.60, t_s_ = 0.49) and males (CL_inf_ = 46.0 mm, K = 1.10 year⁻^1^, and C = 0.64, t_s_ = 0.45) were derived from mean length-at-relative-age estimates under the seasonal growth assumption, using previously described methods. The graphical representations of these von Bertalanffy growth curves are shown in Figure 8. The growth curve exhibited seasonal oscillation, with a growth coefficient (C) of 69% for females and 62% for males, and the phase of slow growth (WP) occurring around December.

### 3.7. Selectivity and Mortality

**Figure 9:**
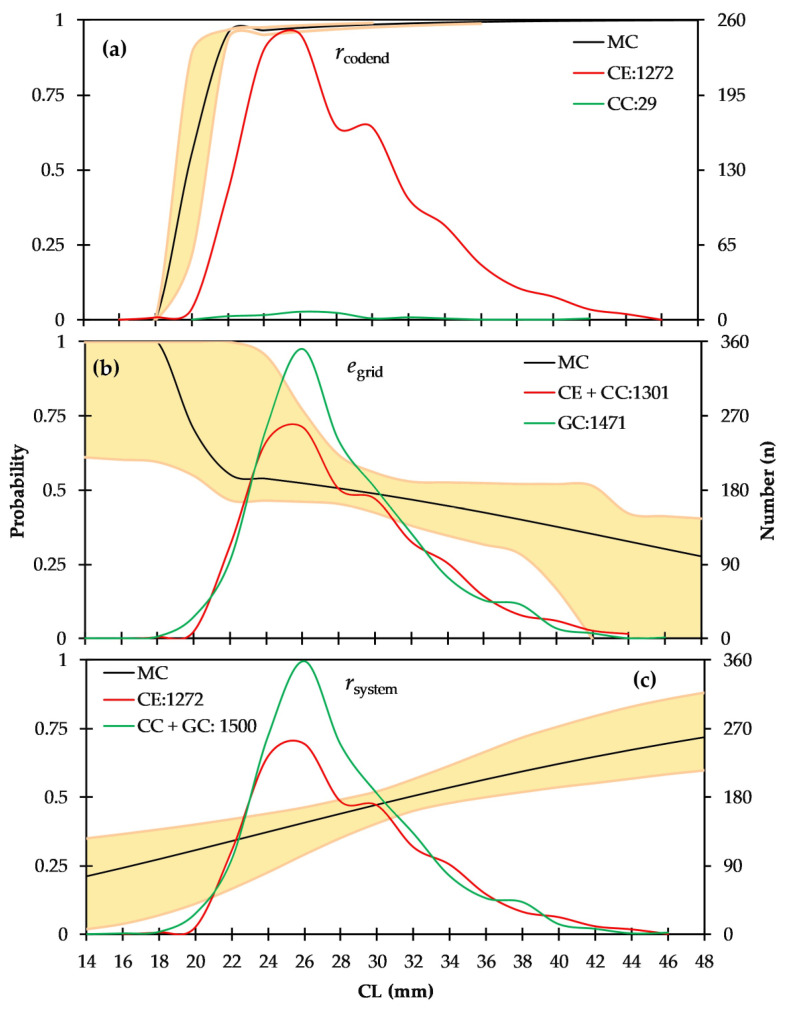
The selection curve for *Penaeus aztecus*, represented by the thick logistic line with confidence intervals, is derived from pooled data. The observed retention values are shown as red circles, while the size structures of entered specimens are depicted by the thick line and escaped specimens by the broken line. (**a**) Retention probability of the codend. (**b**) Escapement probability of the TED grid. (**c**) Retention probability of the entire system.

During the hauls, 100% of five *C. caretta* and 65% of 101 individuals from eight elasmobranch species were excluded from the TED escape hole. The 44 mm DM codend exhibited poor selectivity for *P. aztecus*, with a 19.9 mm CL_50%_ and SR of 0.6 mm. Of the shrimps retained in the codend, 47% were classified as young-of-the-year specimens (CL < 28 mm), and 83.1% were smaller than the FMS (Figure 9a). Only 3.1% of the shrimps entering the codend were able to escape. Of the shrimps entering the trawl, 53.1% were excluded by the TED grid. Meanwhile, 92% and 50% of the excluded shrimps were smaller than the FMS and below the recruitment size threshold, respectively. Although the CL_50%grid_ increased to 28.8 mm, the large SR_grid_ (30 mm) allowed a wide size range of shrimp to pass through the grid into the codend (Figure 9b). Statistical analysis showed no significant difference between CL_50%grid_ and CL_50%system_, as the confidence intervals of both values overlapped. The combined system selectivity resulted in 31.6 mm CL_50%_ (Figure 9c). A selectivity curve that transitions from sigmoid to flat would increase both the escape rates of large individuals and the capture rates of small individuals (Table 5).

The total mortality rate (Z) for male shrimps ranged monthly from 0.026 to 0.658 month⁻^1^ (mean: 0.343 month⁻^1^), and for female shrimps, it varied from 0.014 to 0.100 month⁻^1^ (mean: 0.320 month⁻^1^). Both rates showed a decreasing trend from July, when population density was at its peak, to May, the last month before new recruitment occurred. Using the “exponential decay model”, the percentage of survivors in the male and female populations decreased significantly by September, coinciding with the opening of commercial bottom-trawl fisheries, to 36.8% and 58.5%, respectively. By May, the survivor percentage dropped further, with only 3.6% of the population remaining.

## 4. Discussion

Despite the rapid expansion of *P. aztecus* along the northeastern Mediterranean coastline [11] within 15 years of its first finding in Antalya Bay [10], a lack of data on the species’ population dynamics makes this study especially important. This study provides the first comprehensive insights into the biological patterns of *P. aztecus* in the Mediterranean, aiding in more accurate assessments of potential stocks in the basin’s various seas.

“Since the actual spawning event by brown shrimp has not been observed in situ, statements regarding the site and time of spawning are based upon the capture of eggs, larvae, or spent adults [36]”. Unfortunately, nearly four decades later, these approaches remain unchanged.

*P. aztecus* is reproductively active year-round in the South Atlantic region from North Carolina to northeast Florida [1,7,25,37,38,39,40], with some authors [37,41] suggesting a single peak breeding period from February to March along the southeastern Atlantic coast and in Laguna Madre, while others propose two peaks in the northern Gulf of Mexico: one from September to November [7] and the second from April to May [25] or April to June [7]. In the northwestern Gulf of Mexico, the peaks occur in late spring and autumn [2,39]. Most Gulf of Mexico studies estimate *P. aztecus* reproductive timing using the recruitment of post-larval juveniles to marshes in lagoons and 2–3 month back-calculations. The recruitment of tiny shrimp occurs primarily from June to August, indicating that the generation was likely born between March and May [42]. In Antalya Bay in the eastern Mediterranean, maturity and spawning occur in successive months, with peak intensities in summer (June–July) and autumn (September–October) (Figure 4a,b). However, the year-round presence of females in Stage III and IV indicates that low-level spawning continues.

The number of ovarian maturity stages for *P. aztecus* in the studies conducted so far (only three) is inconsistent, with proposed classifications of seven stages [25], three stages [26], and seven stages in experimental aquaculture trials in Polynesian waters [43]. However, only Brown and Patlan [26] provided visual evidence to support the developmental stages and their explanations. In our study, to facilitate the macroscopic determination of stages in future research, a four-stage key (Figure 3) for gonadal maturity of *P. aztecus* was developed based on previous studies and used very easily in our gonadal determinations. This scheme is visually supported and provides a framework for classifying gonadal maturity. In the Gulf of Mexico, female *P. aztecus* reach maturity between 140 mm TL (≌35 mm CL) and 153 mm TL (≌37 mm CL) [2,30]. The first maturation size (FMS) for females was estimated to be 36 mm CL in Antalya Bay, with the minimum size of mature females observed at 30 mm (Figure 4d). The results from the eastern Mediterranean align with those observed in the Gulf of Mexico.

The demersal eggs of *P. aztecus* develop into larvae within 24 h and become planktonic, undergoing several developmental stages as they are transported to the coast by tidal and wind-driven currents [36]. Post-larvae, around 7–9 mm [8] and approximately 40 days old, enter estuaries and move into marshes to settle in preferred habitats, where they feed and grow rapidly [44]. A review of literature on the timing of estuarine recruitment of *P. aztecus* in the Gulf of Mexico reveals considerable variations. Some studies propose year-round recruitment [45,46], while others identify a peak between April and May [40,47]. Additionally, some document recruitment in February [41,48], and others report a longer recruitment period from mid-December to early May [33,40]. *Farfantepenaeus* species emigrate from estuaries during their sub-adult stage (CL ≥ 15.0 mm) [49], but smaller sizes remain resident for about three months. Shrimp return to offshore waters as sub-adults, typically at sizes of 80–100 mm TL (≌18–23 mm CL) [9,48,50,51]. Marine recruitment in Mexican brown shrimp populations typically occurs at 4 months [52], after which water temperatures or crowding trigger offshore emigration [38]. However, some studies [53,54] suggest that juveniles of *P. aztecus* may also use open bays as nursery areas. In Antalya Bay, marine recruitment begins with a few individuals in June (mean modal CL of 20–26 mm), with 68% of annual recruitment occurring in summer months. Recruitment of smaller groups continues through autumn. The modal length of early summer recruits reached 32 mm CL (for females) in November, while a bimodal group that missed summer growth had a mean CL of only 22 mm. The smallest recruit specimens observed over 27 months were 14 mm CL for both sexes. In Antalya Bay, the Beşgöz Stream (approximately entrance width 40 m, maximum width 90 m, 3 km long) and the Acısu Stream (approximately entrance width and maximum width 60 m, 2.5 km long) are two small and narrow lagoonal formations characterized by reeds and meadows. A previous study [11] hypothesized that post-larval and juvenile *P. aztecus* might utilize these streams as feeding grounds in Antalya Bay, based on information obtained from local fishermen. The entering of small shrimp in these lagoonal areas begins in May, peaks in July and August, then declines with the onset of rainfall toward the end of October (personal communication with Yıldırım Kındı (head of the fishermen’s cooperative) on 20 November 2024). As reported, fishermen in the Damietta Nile estuary, Egypt, collected 700–800, 400–600, and 150–200 shrimp per kilogram during February–March, March–April, and May 2017, respectively [13]. This finding supports the validity of our fishermen’s claims regarding shrimp entering the estuary in Antalya Bay.

Nearly all published growth studies of *P. aztecus* focus on post-larvae and juveniles in estuarine environments [36,39,42,52,55,56,57], which are easier to study and provide data useful for predicting commercial harvests [55]. It was emphasized that length–frequency data are highly susceptible to biases caused by gear selectivity (e.g., small fish escaping through the meshes of commercial fishing gear), migration (e.g., juveniles aggregating in specific nursery areas before gradually migrating to fishing grounds), and sampling errors, all of which can result in overestimations or underestimations of cohort mean lengths [58]. Despite this, most growth studies on *P. aztecus* [38,40,42,57,59,60] rely on length data from commercial fisheries (Table 6). For instance, analyzed shrimp landed at canneries from offshore industrial fisheries and estimated that female *P. aztecus* reached sizes of 108 mm TL (≌23 mm CL) at 3 months, 157 mm TL (≌35.2 mm CL) at 6 months, and 206 mm TL (≌47 mm CL) at 12 months [42]. An analysis of fisheries-dependent data from the Laguna Madre identified a maximum modal group age of 16 months [37], with male individuals exhibiting age classes ranging from 2 to 13 months and lengths of approximately 22–41 mm CL, while females ranged from 2 to 15 months, with lengths of approximately 21–54 mm CL [42]. Growth slows significantly once shrimp reach 100 mm TL (≌23 mm CL), with males generally growing more slowly than females [1], and an average lifespan of about 18 months reported by [61], although many females are likely to live longer [50]. Most adults are believed to spawn only once [45] and appear to die shortly afterward, completing an annual life cycle [55]. However, tagging studies suggest that some individuals may live up to 2.5 years or more [36]. In our study, the pooled data from a 27-month period revealed that female individuals exhibited a mean growth rate of 1.5 mm CL per month, culminating in a mean length of 41.5 mm CL at one year after marine recruitment. A similar growth trajectory was observed in the male individuals, with an average monthly growth of 1.3 mm CL, resulting in a length of 35.8 mm CL. The oldest recorded individuals, relative to their age following marine recruitment, were 21 months old for females and 14 months old for males. Notably, less than 5% of individuals survive to reach their second year of life after marine recruitment.

As the present study did not cover the period from spawning to marine recruitment, several questions remain unanswered: (*i*) Twelve months after marine recruitment (June to June), if females grow from 23 mm CL to 41 mm CL (an increase of 18 mm) and males grow from 20 mm CL to 36 mm CL (an increase of 16 mm), how many months old were shrimps at the time they reached the marine recruitment size? (*ii*) Although the maximum spawning peaks of *P. aztecus* occur in summer and autumn, how is it that nearly 70% of the annual marine recruitment takes place during the same summer, particularly in July and August? In other words, which spawning period leads to this intense recruitment in summer?

A reference growth value of 0.6 mm/week was identified on the basis of growth rates of 0.49–0.94 mm CL/week for 8–21 mm CL shrimps [57], and an average of 0.6 mm CL/week for 11–29 mm CL shrimps [52] in Gulf of Mexico lagoons. When applied to Antalya Bay, it is estimated that approximately 9 months would be required for female individuals to reach a recruitment size of 23.5 mm CL (23.5 mm ÷ 0.6 mm = 39.1 weeks ≈ 9 months). Laguna Madre is a hypersaline and shallow lagoon (averaging only 1.1 m deep and 4 to 6 miles wide) that provides critical growth habitat for juvenile and sub-adult shrimp along the western coast of the Gulf of Mexico. It extends 144 miles along the U.S. border and another 126 miles into Tamaulipas, Mexico [62]. In contrast, the habitats of Antalya Bay (eastern Mediterranean) differ significantly. Here, water depth increases rapidly, reaching 5 m within 0.5 km from shore and 20 m at 2.5 km. Estuarine areas in Antalya Bay are also very limited. Narrow rivers, such as the Acısu and Beşgöz, provide migratory habitats for juveniles, but they are only 50–250 m wide and extend a few kilometers inland. Moreover, the water temperature and salinity in these estuarine habitats are markedly lower than those in the Laguna Madre. Long-term averages (2005–2020) of water temperature and salinity at two stations on the Acısu River (at the river mouth and 1 km upstream) were reported as 21.5 °C and 20.8 °C for temperature, and 4.362 ppt (7888 μS/cm) and 2.39 ppt (4489 μS/cm) for salinity, respectively [63]. However, the growth rates of juveniles and sub-adults in estuarine habitats have been positively correlated with water temperature [52,55,56,57] and salinity levels above 38‰ [52,64]. Laboratory rearing studies on *P. aztecus* juveniles demonstrated an increase in TL of 1.3 mm per week at a salinity of 33‰, which rose by 60% to 2.1 mm per week at a salinity of 38‰ [64]. Juvenile brown shrimp growth slows significantly at temperatures below 18 °C [2]. When the data indicating that individual weights reached 5 g and 6.7 g (150–200 shrimp per kg) during the last collection month (February to May) for cultivation in the Nile estuary, Egypt [13] were applied to the TW/CL equation, it was estimated that the CL of individuals in May ranged between 16 and 19 mm. In light of this literature, it can be inferred that the growth of *P. aztecus* individuals in Antalya Bay prior to marine recruitment is slower, resulting in a longer residence time in the estuary compared to their counterparts in the Gulf of Mexico. Consequently, the duration from spawning to marine recruitment may extend to 12 months in Antalya Bay. However, the most accurate answers to these questions will require further studies specifically focusing on the period from spawning to marine recruitment.

The following parasites were recorded by various authors in *P. aztecus* populations in the Gulf of Mexico [53]: *Nematopsis penaeus* (Sprague, 1954) has been found in the intestinal tract, *Cephalolobus penaeus* (Kruse, 1959) in the stomach filters, *Nosema nelsoni* (Sprague, 1950) in muscle tissue throughout the body, *Prochristianella penaei* (Kruse, 1959) and *Contracaecum sp*. in the digestive gland and stomach, and *Zoothamnium* sp. on the gills of *P. aztecus* populations in the Gulf of Mexico. However, no information regarding the infestation of the parasitic isopod *E. ingens ingens*, which is reported in this study, was found in the Atlantic population. One of the most common bopyrid isopods, *E. ingens ingens* [65], native to the Indo-West Pacific Ocean [66], was first described by Nobili [67] on *P. semisulcatus* from the Red Sea and has since been reported in 11 penaeid host species [68]. Female bopyrid isopods release epicaridium larvae that parasitize calanoid copepods as intermediate hosts, later transforming into cryptoniscus larvae, which settle in the branchial chambers of crustacean hosts [69]. Parasitism imposes a metabolic burden on the decapod host, as female *E. ingens ingens* feed on hemolymph and ovarian fluids after piercing the host’s inner cuticle [70]. Its effects on the host are diverse and often severe, including reduced fecundity, parasitic castration, and reduced moulting frequency [71]. Although the first record of *E. ingens ingens* in the Mediterranean was from the Mersin Gulf, Turkey [72], more than half a century later, this bopyrid species was found infecting *P. aztecus* in the Antalya Gulf as the 12th penaeid host [73]. Deval and Koçancı [11] analyzed raw data from the first survey of this study, detailing infestation ratios by sex and month in *P. aztecus*, as well as the size at first accompaniment and maturity of female parasites, along with their eggs and larvae. Galil et al. [15] say that the introduction of parasites with the host *P. aztecus* could be a threat to native biodiversity and economic value of Penaeid populations elsewhere in the Mediterranean Sea. Interestingly, although *P. semisulcatus* was first detected as a host for *E. ingens ingens* and has been found to be the most commonly parasitized shrimp species by this bopyrid [66], no infection was detected in the other six penaeid species (*P. pulchricaudatus*, *M. monoceros*, *P. kerathurus*, *M. aegyptia*, and *P. hathor*) in Antalya Bay (except for a single infected female *P. semisulcatus*) [11], where these species constitute 46.6% of the shrimp composition. Owens and Glazebrook [74] suggested that the cryptonisci larvae of *E. ingens ingens* selectively search for the host prawn *P. semisulcatus* and only infest other shrimp species if *P. semisulcatus* is not found within a reasonable period of time. However, the fact that *P. semisulcatus* constituted 11% of the shrimp composition during the sampling period invalidates the suggestion of Owens and Glazebrook [74]. Gopalakrishnan et al. [75] offer an interesting suggestion that the higher infection rate of another bopyrid isopod, *Palaegyge buitendijki* (Horst, 1910), in female rather than male prawns (*Palaemon malcolmsonii* H. Milne Edwards, 1844) may be due to females having more fatty acid reserves for reproductive purposes. As Gopalakrishnan et al. [75] said, it might be that the chemical composition of the *P. aztecus* may affect its selectivity. The precise rationale behind the parasite’s selective behavior remains to be fully elucidated at this juncture; nonetheless, it is speculative but possible that the presence of *P. aztecus* within the ecosystem exerts a significant influence on the parasite’s species preference, potentially resulting in a lower prevalence or no infection in other penaeid species.

In the U.S., the use of TEDs became mandatory in offshore and inshore shrimp fisheries in 1987 and 1992, respectively, ensuring that 97% of all sea turtles caught in shrimp trawls were able to escape, while the 51 mm bar spacing grids in 2010 found they reduced the capture of sharks and rays by approximately 80% [76]. In the Mexican offshore shrimp fishery, TEDs have been required since 1993 to reduce sea turtle mortality, with codend mesh sizes typically ranging from 38 to 45 mm [77]. Although two species of sea turtles, *C. caretta* and *Chelonia mydas*, nest intensively along the central and eastern Mediterranean coasts and are incidentally caught in bottom trawl fisheries, no Mediterranean countries have yet made the use of TEDs mandatory in bottom trawl fisheries. In the study, TEDs excluded 100% of five loggerhead turtles (*C. caretta*) and 65% of 101 individuals of eight elasmobranch species. However, the 44DM codend, which is legally used in the commercial fishery, demonstrated significantly poor selectivity for *P. aztecus*, with a CL_50%_ of 19.9 mm. The CL_50%_ of the combined system increased to 31.6 mm CL.

Studies in estuarine environments have reported high mortality rates for *F. aztecus* populations, ranging from 0.16 to 0.73 week⁻^1^ [51,57]. Fish predation is the main cause of juvenile shrimp mortality, with additional contributions from environmental factors such as salinity, temperature, and oxygen levels [44]. Findings show even higher mortality percentages in offshore shrimp stocks, with rates of 66% and 81% per month (21% for fishing and 60% for natural causes), respectively [78,79]. Converting the survival percentages from Berry [60] and Klima [61] to total mortality rates using Ricker [34]’s formula gives 1.079 month⁻^1^ and 1.661 month⁻^1^ respectively, reducing survival to only 3% after two months. These rates are substantially higher than the mean monthly rates observed for Mediterranean shrimp stocks in Antalya Bay: 0.343 month⁻^1^ for males (range: 0.027–0.658) and 0.320 month⁻^1^ for females (range: 0.014–1.00). Although a bottom-trawl fishing ban was in effect in the Gulf of Antalya during the two months between July and September, the observed mortality percentage of 58.5% during this period is unlikely to be solely attributable to artisanal trammel-net fisheries in the region. Instead, these high mortality rates are better explained by the natural life cycle of the species, as most females spawn only once [45] and die shortly thereafter [55].

## 5. Conclusions

This study provides the first comprehensive analysis of the population dynamics of *P. aztecus* in the Mediterranean, contributing valuable insights for fisheries management and ecological research. Key findings reveal that spawning occurs year-round in Antalya Bay, with peaks in summer and autumn, aligning with similar patterns observed in the Gulf of Mexico. Growth rates indicate slower development but longer life span compared to Gulf populations, likely due to distinct environmental factors in the Mediterranean. Recruitment is dominated by summer months, suggesting complex spawning–recruitment relationships requiring further investigation. Additionally, this study introduces a practical four-stage gonadal maturity classification to support future research. Significant mortality factors include predation and fishing, highlighting the need for sustainable management practices. The absence of TEDs in Mediterranean fisheries emphasizes a critical gap in conservation efforts, particularly for reducing bycatch of endangered species. Continued research is essential to address gaps in early life history and recruitment dynamics. Therefore, the spawning and recruitment dynamics of shrimp species in the Mediterranean Sea are vulnerable to a range of environmental stressors associated with climate change [80,81] and habitat degradation. While certain species may temporarily benefit from warmer conditions, the cumulative impacts of ocean warming, acidification [82], hypoxia [83], habitat (seagrass meadows and estuarine areas) loss [84], and salinity changes [85] are likely to have negative consequences for long-term population sustainability. Adaptive management strategies, habitat restoration efforts, and continuous monitoring are crucial for mitigating these impacts and supporting the resilience of shrimp populations in the Mediterranean.

## Figures and Tables

**Table 1:** List of observed and calculated dates for each sampling month during the surveys, including haul duration (HD, minute), swept area (SA, km^2^), biomass (BI, kg. km^−2^), total density (D_TOT_, ind. km^−2^), density of females (D_F_, ind. km^−2^), density of potential spawner females (D_FPS_, ind. km^−2^), density of mature females (D_FMAT_ ind. km^−2^), and density of recruits (D_REC_, ind. km^−2^). Also provided are average carapace length (mCL, mm), average total weight (mTW, g), monthly average sea surface temperature (SST), and moonlight percentage (MoL %). ‘ns’ indicates non-studied parameters.

	Date	HD (min)	SA (km^−2^)	Catch (kg)	BI	D_TOT_	D_F_	D_FPS_	D_FMAT_	D_REC_	% Infection	mCL (mm)	mTW (n/g)	SST(°C)	MoL (%)
Survey 1	27 March 2019	120	0.15	2.44	16.5	311	162	142	14	7	84.8	38.7	38.1	17.3	63.0
	17 April 2019	110	0.16	1.11	7.0	215	108	95	32	0	61.8	35.9	32.6	17.9	90.8
	5 May 2019	105	0.15	1.01	6.7	198	92	86	66	0	46.7	36.3	32.6	21.6	90.4
	21 June 2019	50	0.15	1.14	7.8	303	110	96	62	62	11.4	31.3	25.9	26.3	88.0
	20 July 2019	160	0.23	16.39	70.9	5559	2321	169	134	3609	0.9	26.5	12.7	27.7	91.9
	28 August 2019	180	0.23	10.09	43.6	3431	1832	1171	1093	774	15.2	30.9	12.7	29.0	9.6
	13 September 2019	60	0.12	5.22	44.5	2523	1210	281	153	1347	27.4	27.3	17.6	28.5	98.5
	8 October 2019	60	0.09	1.59	17.4	1935	1028	448	164	383	48.6	30.0	20.1	26.1	70.6
	28 November 2019	110	0.19	4.24	22.1	814	376	266	120	125	55.1	32.1	27.2	23.2	1.9
	20 December 2019	90	0.14	1.33	9.4	330	239	190	84	35	34.0	33.6	28.3	19.9	42.4
	26 January 2020	120	0.19	1.72	9.0	314	157	99	10	26	66.7	33.1	28.7	18.3	1.0
	26 February 2020	120	0.19	1.27	6.6	188	104	73	16	10	72.2	34.2	35.3	17.1	0.1
	14 June 2020	150	0.21	0.56	2.6	80	42	14	5	19	35.3	34.5	32.9	23.7	44.3
	16 July 2020	145	0.19	6.90	36.2	1911	808	89	79	1029	2.2	27.3	19.0	27.0	23.7
Survey 2	24 November 2020	138	0.19	1.42	7.6	344	183	21	11	215	23.4	25.8	22.2	23.6	66.1
	24 December 2020	170	0.27	3.72	13.8	569	324	134	100	127	28.8	30.5	24.3	20.1	67.4
	21 January 2021	185	0.29	1.32	4.6	191	94	45	21	21	47.3	35.4	24.0	18.9	49.9
	21 February 2021	180	0.31	1.91	6.2	201	81	55	39	10	41.9	32.2	30.8	17.9	50.9
	20 March 2021	145	0.21	1.27	6.0	199	123	95	33	5	47.6	33.8	30.2	17.9	33.8
	19 April 2021	180	0.26	0.71	2.8	90	39	39	12	0	52.2	34.2	32.3	18.4	36.3
	18 May 2021	175	0.25	0.83	3.4	90	41	41	24	0	^ns^	37.5	37.7	22.3	30.7
	16 June 2021	200	0.28	3.27	11.6	623	345	75	43	420	^ns^	26.6	18.7	24.3	26.5
	8 July 2021	175	0.25	8.92	35.9	2647	1110	0	0	2144	^ns^	24.7	13.6	27.2	1.3
	19 August 2021	170	0.24	10.73	44.8	2541	1325	288	54	1346	^ns^	26.6	17.6	29.5	84.0
	25 September 2021	150	0.18	4.55	25.8	1627	958	227	108	1043	^ns^	25.4	15.9	28.7	85.8
	21 October 2021	160	0.24	0.97	4.0	276	119	25	16	194	^ns^	25.5	14.5	25.3	99.9
	12 November 2021	190	0.27	1.89	7.0	556	256	22	15	419	^ns^	24.6	12.6	22.3	53.7

**Table 2:** Results of one-way ANOVA for log10-transformed biomass (BI), abundance indices (total density (D_TOT_), density of recruits (D_REC_), and density of mature females (D_MAT_)), and other important parameters (sex ratio (SR), infection percentage in total (IR), infection percentage in potential female spawners (IR-F_PS_), mean CL, and TW) of *P. aztecus* across different seasons, surveys, and months. The degrees of freedom (df) are presented as between groups, within groups, and total, respectively. (Bold *p* value denotes significant difference in variables).

Factors	Dependents	df	F	*p*	Factors	Dependents	df	F	*p*
Season	Log D_TOT_	3/25/28	8.544	**0.000**	Survey	Log D_TOT_	1/22/23	0.677	0.419
	Log BI	3/25/28	4.616	**0.011**		Log BI_TOT_	1/22/23	2.003	0.171
	Log D_REC_	3/25/28	9.136	**0.000**		Log D_REC_	1/22/23	0.009	0.927
	Log D_MAT_	3/25/28	0.540	0.659		Log D_MAT_	1/22/23	3.972	0.059
	SR	3/25/28	0.876	0.467		SR	1/22/23	0.074	0.788
	Mean CL	3/23/26	16.184	**0.000**		Mean CL	1/22/23	2.549	0.125
	Mean TW	3/25/28	13.446	**0.000**		Mean TW	1/22/23	0.805	0.379
	IR	3/18/21	7.606	**0.002**		IR	1/15/16	0.000	0.989
	IR-F_PS_	3/22/26	1.098	0.371		IR-F_PS_	1/21/23	0.230	0.636
Month	Log D_TOT_	11/17/28	8.054	**0.000**					
	Log BI	11/17/28	5.969	**0.001**					
	Log D_REC_	11/17/28	2.519	**0.042**					
	Log D_MAT_	11/17/28	0.942	0.527					
	SR	11/17/28	1.014	0.475					
	Mean CL	11/15/26	4.516	**0.004**					
	Mean TW	11/17/28	6.313	**0.000**					
	IR	11/8/19	2.549	0.097					
	IR-F_PS_	11/14/25	1.201	0.367					

**Table 3:** Results of Spearman’s rho correlations for log10-transformed biomass (BI), abundance indices (total density (D_TOT_), density of recruits (D_REC_), and density of mature females (D_MAT_)), and other important parameters (sex ratio (SR), infection percentage in total (IR), mean CL, and TW) of *Penaeus aztecus* with sea surface temperature (SST) and moonlight (%). (Bold *p* values indicate statistically significant relationships).

			Spearman’ Rho	
Independents	Dependents	N	*r*	*p*
SST	Log D_TOT_	27	0.724	**0.000**
	Log BI	27	0.631	**0.000**
	Log D_REC_	27	0.763	**0.000**
	Log D_MAT_	27	0.459	**0.016**
	SR	27	−0.162	0.421
	Mean CL	27	−0.677	**0.000**
	Mean TW	27	−0.741	**0.000**
	IR	20	−0.794	**0.000**
Moonlight	Log D_TOT_	29	0.102	0.597
	Log BI	29	0.105	0.586
	Log D_REC_	29	0.072	0.711
	Log D_MAT_	29	0.278	0.144
	SR	29	−0.125	0.518
	Mean CL	27	−0.175	0.384
	Mean TW	29	−0.202	0.292
	IR	22	−0.303	0.170

**Table 4:** Means (±SD) of carapace length (CL), total weight (TW), gonad weight (GW), and gonadosomatic index (GSI) across the four ovarian maturation stages of *Penaeus aztecus*.

Variables	St.I (59)	St.II (117)	St.III (77)	St.IV (63)
CL (cm)	34.5 ± 3.1 *	38.1 ± 4.3	38.4 ± 4.9	38.7 ± 3.8
TW (g)	29.4 ± 7.5 *	37.4 ± 11.4	39.1 ± 14.3	40.2 ± 10.2
GW (g)	0.0936 ± 0.062 *	0.3197 ± 0.216 *	0.7956 ± 0.361 *	1.7737 ± 0.843 *
GSI	0.312 ± 1.59 *	0.8315 ± 0.396 *	2.0262 ± 0.559 *	4.3666 ± 1.499 *
Range of GSI	0.072–0.78	0.137–2.081	1.003–3.964	2.254–7.688

Sample size is presented in parentheses, and * indicates a significant difference (*p* = 0.05) from other stages.

**Table 5:** Mean selectivity results and fit statistics for the escapement ahead of the grid tested during the sea trials. Values in brackets represent the 95% CI from the Efron percentile bootstrap. Df indicates the degrees of freedom.

Parameters	Values
CL50%_grid_	28.8 (20.6–42.4)
SR_grid_	30.3 (14.6–82.8)
C_grid_(%)	65.6 (24.4–100.0)
CL50%_codend_	19.9 (19.6–20.3)
SR_codend_	0.56 (0.11–1.12)
CL50%_system_	31.6 (28.7–35.5)
SR_system_	34.5 (15.7–72.0)
Df	14
Deviance	12.8
*p*-value	0.5429

**Table 6:** Growth and mortality parameters of *Penaeus aztecus* estimated in the Atlantic and Mediterranean.

		TW/CL		Length Range (mm)		Parameters of VBGF					Mortality (per Month)			
Data	Sex	a	b	TL	CL	TL_inf_	CL_inf_	K	to	MMAG	F	Z	Refer.	Area
MR	M	0.00082	2.94		15–40		30	0.32 ^m^	−5.98 ^w^		0.21	0.57	[60]	U.S.
MR	F	0.00113	2.84		15–40		36	0.17 ^m^	−7.2 ^w^					
FI	M	11.6 × 10^−6^	2.91		10–48								[59]	
FI	F	9.5 × 10^−6^	2.94		12–57									
FI	B			25–200	6–47 ^c^							0.16–0.73 ^w^	[57]	Mexico
FD	M	0.00021	2.32	93–172	22–41 ^c^	178	42 ^c^	0.26 ^m^	−0.239 ^m^	15			[42]	
FD	F	0.00001	2.97	91–216	21–50 ^c^	236	59 ^c^	0.16 ^m^	−0.759 ^m^	13				
FD	B			40–130	9.4–30 ^c^	204	48 ^c^	0.26 ^m^	−0.292 ^m^	16	0.11–0.65		[38]	
FD	B									16	0.5	0.74	[40]	
FI	M	0.0038	2.522		14–44		45.4	1.31 ^y^	−0.12 ^y^	15		0.33	This study	Medit.
FI	F	0.0035	2.546		14–64		56	1.11 ^y^	−0.14 ^y^	21				

MR: marc-recapture; FD: fisheries-dependent; FI: fisheries-independent; MMAG: maximum modal age group per month; Medit.: Mediterranean; ^w^: week^−1^; ^m^: month^−1^; ^y^: year^−1^; ^c^: converted using the TL/CL regression formula by [55].

## Data Availability

The raw data supporting the conclusions of this article will be made available by the corresponding author on request.

## Supplementary Materials

The following supporting information can be downloaded at https://www.mdpi.com/article/10.3390/ani15040561/s1, Table S1: Standardized length–frequency data (LFSD, ind. km^−2^) of *Penaeus aztecus* for both sexes, obtained over a 27-month period during sampling in the eastern Mediterranean (1S: first survey, 2S: second survey); Table S2: Pooled standardized length–frequency distribution (ind. km^−2^) for female *Penaeus aztecus* from two surveys; Table S3: Pooled standardized length–frequency distribution (ind. km^−2^) for male *Penaeus aztecus* from two surveys.
