# Supplementary material for: Population Dynamics of the Widespread Alien Decapod Species, Brown Shrimp (Penaeus aztecus), in the Mediterranean Sea
Source: Animals (Basel). 2025 Feb 14;15(4):561. doi: 10.3390/ani15040561 (PMC11851461; doi:10.3390/ani15040561)
Supplement: Supplementary file 1 [file animals-15-00561-s001.zip › Deval and Deniz_Supplementary Table S2.pdf]

Supplementary Table S2. Pooled standardized length-frequency distribution (ind.km^{-2}) for female *Penaeus aztecus* from two surveys.

CL (mm)	Jun	Jul	Aug	Sep	Oct	Nov	Dec	Jan	Feb	Mar	Apr	May
14			8		5							
16			13	11	5							
18	4	2	44	26	5	2						
20	18	16	21	65	8	11						
22	60	61	38	55	16	22		2				
24	34	243	48	116	16	13	9	2				
26	14	403	57	160	37	25	19	3	2	2		
28	7	364	150	142	68	29	18	5	2	2		
30	5	354	227	129	63	38	28	10	11	5		
32		189	243	125	53	32	46	10	14	15	3	3
34		32	321	95	83	46	63	35	14	15	7	9
36	14	17	210	75	42	38	24	25	13	45	20	9
38	21	24	123	64	29	35	43	14	11	33	26	9
40	19	6	63	13	11	16	23	14	14	17	3	7
42	19	4	9	3	11	5	4	5	8	13	6	25
44	12		4	5		4	5		4		3	5
46											2	2
48												
50										3		
52										10		
54										3		
56										3		
Σ=	221	1716	1579	1084	688	253	281	126	93	163	71	70
