# Supplementary material for: Population Dynamics of the Widespread Alien Decapod Species, Brown Shrimp (Penaeus aztecus), in the Mediterranean Sea
Source: Animals (Basel). 2025 Feb 14;15(4):561. doi: 10.3390/ani15040561 (PMC11851461; doi:10.3390/ani15040561)
Supplement: Supplementary file 1 [file animals-15-00561-s001.zip › Deval and Deniz_Supplementary Table S3.pdf]

Supplementary Table S3. Pooled standardized length-frequency distribution (ind.km^{-2}) for male *Penaeus aztecus* from two surveys.

CL (mm)	Jun	Jul	Aug	Sep	Oct	Nov	Dec	Jan	Feb	Mar	Apr	May
14		0	2									
16		0	29	11								
18	9	28	44	88		2						
20	28	80	38	104	8	30						
22	27	524	47	160	55	40	2					
24	8	908	183	200	109	46	9	3	4			
26	2	611	488	192	133	48	42	14	4	3		
28	12	145	383	115	103	40	53	21	23	11	4	7
30	9	39	142	94	48	23	35	40	20	45	15	7
32	18	4	35	17	55	22	17	26	23	22	19	9
34	10	15	4	4		16	9	18	21	15	15	21
36	10	17	4			16	2	3	6	16	15	24
38	8	4	6			2		2			5	5
40	1	0	2								3	5
42											3	
Σ=	142	2376	1408	985	510	283	168	127	102	112	79	77
