# Supplementary material for: Population Dynamics of the Widespread Alien Decapod Species, Brown Shrimp (Penaeus aztecus), in the Mediterranean Sea
Source: Animals (Basel). 2025 Feb 14;15(4):561. doi: 10.3390/ani15040561 (PMC11851461; doi:10.3390/ani15040561)
Supplement: Supplementary file 1 [file animals-15-00561-s001.zip › Deval and Deniz_Supplementary_Table S1.pdf]

Supplementary Table S1. Standardized length-frequency data (LFSD, ind.km^{-2}) of *Penaeus aztecus* for both sexes, obtained over a 27-month period during sampling in the eastern Mediterranean (1S: first survey, 2S: second survey).

		2019											2020					2021													
	CL (mm)	M	A	M	J	J	A	S	O	N	D	J	F	J	J	N	D	J	F	M	A	M	J	J	A	S	O	N	1S	2S	All
Female	14								0																17					17	17
	16								11																25	23			11	48	59
	18								11							4							7	4	88	51		4	11	154	168
	20				14	4		34	0							4							28	28	42	96	16	22	52	233	289
	22				34	65	13	43	11							7		3					114	56	63	68	21	44	166	369	543
	24				14	233	26	136	33					4	4	19	19	3					68	253	71	96	21	26	442	557	1025
	26				14	484	43	196	66	5				7	97	19	37	7	3	5			28	322	71	125	8	44	808	651	1582
	28				7	519	82	187	131	21	14				156	26	22	10	3	5			14	209	217	96	4	37	961	619	1763
	30					571	203	196	153	31	7	5	16		141	11	48	14	6	9			11	137	251	62	16	44	1182	600	1934
	32	20	6	7		277	294	136	273	52	28	10	16		112	22	63	10	13	9				101	192	113	8	11	1120	522	1775
	34	20	6	13		65	475	111	262	89	63	52	5		33	7	63	17	23	28	8	4			167	79	12	4	1163	406	1610
	36	61	25	13	21	35	324	77	142	68	14	37	16		19		33	14	10	33	16	4	7		96	74	8	7	832	303	1153
	38	34	44	7	34	48	220	60	98	63	63	21	16		7		22	7	6	14	8	12	7		25	68	4	7	708	182	897
	40	20	6	7	14	13	125	26	44	31	35	21	21	4	0	4	11	7	6	19		8	25						363	77	447
	42	7	13	46	7	9	17		22	10	7	10	10	11	4	4	4		6			4	25			6			159	45	222
	44		6	7	34	0	9			5	7		5	7					3			4	11					4	73	22	103
	46							9													4	4							9	8	17
	48																														
	50	7																											7		7
	52	20																											20		20
	54	7																											7		7
	56	7																											7		7
	Σ=	203	108	99	193	2321	1832	1210	787	376	239	157	104	33	573	127	324	94	81	123	35	41	345	1110	1325	958	119	256	7629	4811	13173
Male	14																								4	11				16	16
	16								0																59	23				81	81
	18				0		9	17	0							4							28	56	79	159		4	26	327	356
	20				34	48	13	111	0					4	4	19							46	113	63	96	16	70	206	405	637
	22				14	605	43	213	77						45	15	4						68	443	50	108	33	104	952	808	1820
	24					1197	56	281	164	37	7	5	5		260	22	11	0	3				25	620	309	119	54	78	1753	1219	3254
	26	7				973	570	315	241	84	28	21	5		320	37	56	7	3				7	249	405	68	25	22	2243	843	3444
	28	14		13	28	233	553	162	186	99	35	31	10		141	15	71	10	36	9	8		7	56	213	68	21	7	1365	507	2028
	30	61	19	13	28	78	259	170	87	57	14	31	10		7	0	56	49	29	28	12				25	17	8	11	829	235	1072
	32	34	25	13	21	9	65	34	109	63	7	42	21	4	4	0	26	10	26	9	12	4	28		4			4	442	124	574
	34	20	19	33		30	4	9	0	47		26	26	7			19	10	16	9	12	8	21		4				215	100	322
	36	14	25	20		35	9		0	47			5	7			4	7	6	19	4	28	21						154	90	251
	38		6	7		9	13			5				4				3			4	4	21						40	33	76
	40		6	7		0	4															4	4						17	8	25
	42		6																										6		6
	Σ=	149	108	106	124	3216	1599	1312	733	438	91	157	83	26	781		112	246	97	120	76	51	49	277	1537	1216	669	156	8116	4794	13829
